# A Note on Trader Sharpe Ratios

**DOI:** 10.1371/journal.pone.0008036

**Published:** 2009-11-25

**Authors:** John M. Coates, Lionel Page

**Affiliations:** 1 Judge Business School, University of Cambridge, Cambridge, United Kingdom; 2 Westminster Business School, London, United Kingdom; Akron University, United States of America

## Abstract

Traders in the financial world are assessed by the amount of money they make and, increasingly, by the amount of money they make per unit of risk taken, a measure known as the Sharpe Ratio. Little is known about the average Sharpe Ratio among traders, but the Efficient Market Hypothesis suggests that traders, like asset managers, should not outperform the broad market. Here we report the findings of a study conducted in the City of London which shows that a population of experienced traders attain Sharpe Ratios significantly higher than the broad market. To explain this anomaly we examine a surrogate marker of prenatal androgen exposure, the second-to-fourth finger length ratio (2D∶4D), which has previously been identified as predicting a trader's long term profitability. We find that it predicts the amount of risk taken by traders but not their Sharpe Ratios. We do, however, find that the traders' Sharpe Ratios increase markedly with the number of years they have traded, a result suggesting that learning plays a role in increasing the returns of traders. Our findings present anomalous data for the Efficient Markets Hypothesis.

## Introduction

Knowing that a trader has made $10 million tells us little about the skill involved in making this money unless we also know how much risk was taken. If this trader could have, with equal probability, lost $100 million then we would have to conclude that the gain of $10 million was merely a bit of luck, even dumb luck. To control for this possibility, trading managers and investors increasingly look at the returns made on capital (in excess of the risk free rate) and then divide by the standard deviation of these returns, giving them a measure known as the Sharpe Ratio [1]. The Sharpe Ratio plays an important role in Modern Portfolio Theory [2], [3], and in the influential Efficient Market Hypothesis [4], [5], [6].

According to this hypothesis market prices provide the best estimate of asset values because they incorporate all available information. Prices change with new information, which by its nature arrives unexpectedly, making the markets random and preventing anyone from consistently outperforming a broad market such as the S&P index or the Dax, the German stock index. The Efficient Market Hypothesis (EMH) does not deny that investors can, through asset allocation decisions, increase the return on their capital, but they can do so only by increasing the amount of risk taken, risk in this case being defined as the standard deviation of returns. If we plot the possible rates of return against the levels of risk needed to achieve them, we should, according to EMH (or at least the Capital Asset Pricing Model, which is the testable prediction of EMH) find a curve that is linear with a slope equal to the Sharpe Ratio of the broad market. EMH implies, therefore, that one can increase one's returns but one cannot systematically increase one's Sharpe Ratio above that of the broad market. We tested this hypothesis by examining the Sharpe Ratios of a group of traders.

To do so we analysed profit and loss (P&L) statements over a 20 month period, between 2005 and 2007, for 53 traders on a trading floor in the City of London. These traders are all male and at the beginning of the study had an average age of 29 years. They engage in what is variously called ‘noise’ or ‘high frequency’ trading, meaning they buy and sell futures contracts on a range of underlying assets, mostly bonds and equities, with the occasional position in currencies or commodities, and hold their positions for short periods of time, usually seconds or minutes. Their trading is proprietary, meaning they trade for their own accounts and do not make markets for clients; they do not therefore benefit from the bid-offer spread on market-making, a lucrative source of profit for what are called ‘flow’ traders at investment banks; nor do they receive fees or commissions of any kind. Lastly, they do not receive a year-end bonus based in part on the performance of the firm as a whole. Their P&L thus derives purely from their trading skill, and this in turn determines their income. Selection acts quickly in this trading environment. Money losing traders do not last long; but the more successful traders on the floor can earn over $10million per year [7].

We calculated monthly Sharpe Ratios for this cohort of traders as well as the broad market. To calculate a Sharpe Ratio one normally calculates the return on invested capital, subtracts the risk-free rate of interest to give the investment's excess return over the risk free rate, and divides by the standard deviation of the returns (Note S1). However, the calculation of a Sharpe Ratio for leveraged traders, like high frequency traders, is slightly different: high frequency traders rarely position trades overnight so do not need to post capital, making it difficult to calculate their rate of return. This does not mean they do not need capital: they must have on hand capital enough to post margin on positions and to cover any trading losses. But that capital, while untouched, is invested in liquid deposits or government bonds and earns on average a rate of interest close to the risk free rate, meaning the return on this capital less the risk free rate nets close to zero [8]. Calculating the Sharpe Ratio therefore reduces to taking mean trading P&L and dividing by the standard deviation of P&L.

## Results

**Figure 1:**
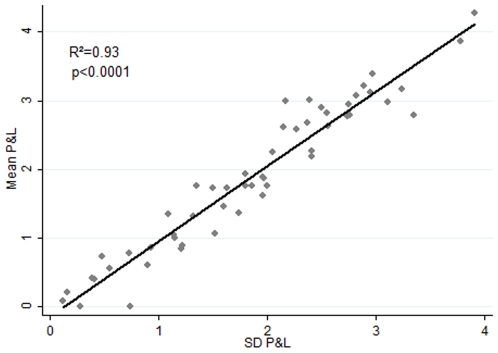
P&L vs. Risk. Plot of traders' P&L against the standard deviation of their P&L. The curve is linear. The intercept is 0, rather than the risk free rate, because returns are zero when the traders do not trade. As P&L data is heavily skewed to the right, we employed a Box-Cox transformation of the P&L to normalise it (SI). The curve is also linear when plotted with raw and log transformed data, as predicted by the Efficient Market Hypothesis.

We began by plotting the traders' P&L against their risk, i.e., the standard deviation of their P&L. We found a curve that is, in accordance with the predictions of EMH, linear (Figure 1). We then calculated the average monthly Sharpe Ratios for the 53 traders and found that it was 0.70.

These traders traded mostly European futures contracts, and specifically the German market, so for comparison we calculated the monthly Sharpe of the Dax, the German Stock index, over the same period to give us an estimate for the broad market. Doing so gave us a monthly Sharpe for the Dax of 0.534. We considered a more comprehensive measure of the broad market, one averaged from both stock and bond markets, perhaps weighted by market capitalisation. However when we looked at the Bund market (German government bonds), the other main market traded by our cohort, we found that bonds were in a bear market over the period, giving Bunds a negative Sharpe ratio of -0.03. We therefore decided to compare the traders against the stronger of the markets they traded – the Dax. Lastly, as our traders were based in London, maintaining their capital and reporting their P&L in pounds sterling, we calculated a Sharpe for the Dax with returns denominated in sterling and found a lower figure of 0.377. To err on the side of conservatism, we again decided for our main statistical analyses to compare our traders against the higher of the Dax Sharpes - the non-currency adjusted Sharpe of 0.534.

**Figure 2:**
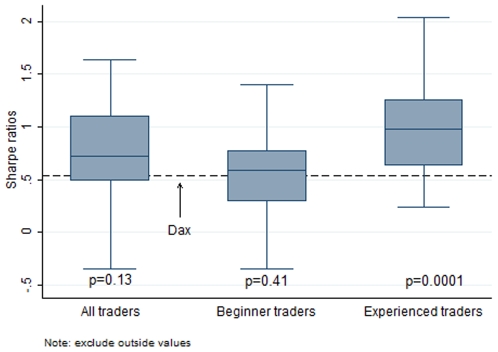
Trader Sharpe Ratios compared to Dax. Box plot showing the distribution of Sharpe Ratios for all traders compared to Sharpe Ratio of the Dax (the German Stock Index); beginner traders only (2 yrs or less); and experienced traders only. T-test p-values indicated below each plot. The Dax Sharpe Ratio over the period is represented as a dashed line.

Using this figure shows us that the traders' average Sharpe of 0.70 was higher than the broad market, although on a first pass the difference was not significant (t-test, p = 0.13, two-tailed, n = 53. Figure 2). We thought it unlikely that the beginner traders among our cohort could either match or outperform the market so we divided the sample into beginner and experienced traders, beginners being defined as any trader who had traded for two years or less [7]. We found that beginner traders averaged a Sharpe Ratio of 0.39, not significantly different from that of the Dax (t-test, p = 0.41, two-tailed, n = 27. Figure 2), although first year traders had a negative Sharpe of -0.04. The experienced traders, however, achieved a Sharpe of 1.02, significantly higher than the Dax (t-test, p = 0.0001, two-tailed, n = 26. Figure 2).

We were concerned, however, that this test did not take into account the fact that the Sharpe Ratio of the Dax is itself a random variable which we observed at a value of 0.534 but which could, over the study period, have been higher or lower. To control for the stochastic nature of the Dax's Sharpe we compared it to the trader Sharpes by means of a bootstrap test. This test consists of the following procedure: we use the observed time series of Dax monthly returns over the period to create new time series of returns by resampling. For each of these new time series we compute a Sharpe Ratio. We can in this way create a distribution of Sharpes by resampling the observed monthly returns of the Dax (Note S1). We can similarly calculate new Sharpes for the traders by resampling their monthly returns. The bootstrap test repeats this process and measures how often the observed Dax Sharpe takes on higher values than the average traders' Sharpe. Our bootstrap tests confirmed our first estimates, that the Sharpe Ratios of the beginner traders were not significantly different from that of the Dax (p = 0.72, n = 27) but that the Sharpes of experienced traders were significantly higher (p = 0.032, n = 26). Running the bootstrap test for the Dax Sharpe denominated in Sterling gives, as expected, even more significant results (p = 0.001, n = 27).

Sharpe Ratios higher than the broad market present an anomaly for the Efficient Market Hypothesis. How can the experienced traders in our cohort outperform the Dax? To answer this question we began by looking at a subset of our sample, n = 47, which had previously taken part in a study which involved measuring a surrogate marker of pre-natal androgen exposure, the second to fourth finger length ratio (2D∶4D) [7]. A lower 2D∶4D, i.e., a longer ring relative to index finger, has been found to correlate with higher levels of foetal testosterone [9], the explanation for the relationship deriving, according to some researchers, from the fact that digit growth and gonadal development are linked by the common influence of the *hoxa* and *hoxd* gene clusters [10], [11]. In our earlier study we found that lower 2D∶4D among high frequency traders predicted higher long term profitability and a greater number of years of survival in the business [7]. We did not attempt to determine just how foetal androgens affect a trader's ability to make money, but we suggested, based on other studies, both animal and human, that the androgens may increase risk preferences [12], confidence, speed of visuo-motor scanning, or physical reactions.

**Figure 3:**
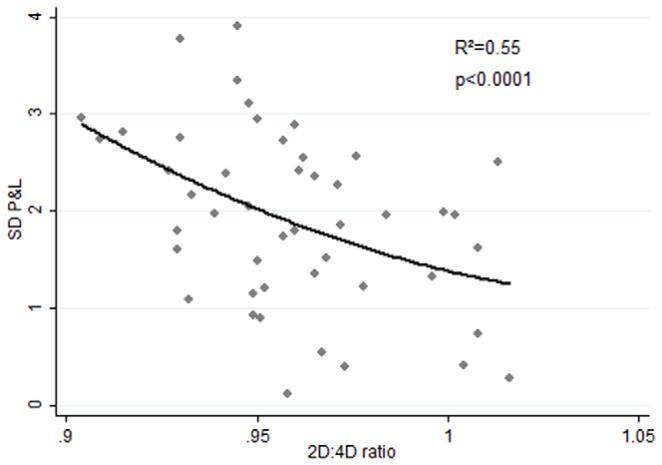
2D∶4D vs risk. Traders' 2D∶4D ratios plotted against their risk, i.e., standard deviation of their P&L, Box-Cox transformed. The fitted curve is quadratic. N = 53.

In the present study we looked into the possibility that higher pre-natal androgen exposure improved the traders' profitability through increased Sharpe Ratios. To conduct this and subsequent analyses the P&L and Sharpe Ratio data were Box-Cox transformed to correct for a pronounced right skew. The correlation between the traders' 2D∶4D and their Sharpe Ratios, however, while displaying the expected sign, only approached significance at the 5% level (n = 47, r = −0.26, p = 0.08). This finding suggested to us that androgens may have their main effect through the amount of risk taken by traders rather than through the amount of money made per unit of risk. We therefore employed robust regression to determine the effect on traders' P&L of i) the standard deviation of their P&L; ii) years of experience; and iii) 2D∶4D. We found that the regression was significant (F test, p<0.00001) and displayed high explanatory power (R^2^ = 0.94). The risk variable was highly significant (p<0.001); as was years of experience (p = 0.004); but 2D∶4D was not (p = 0.911). 2D∶4D was, however, correlated with risk in a simple regression (n = 47, r = −.43, p = 0.001. Figure 3). The low 2D∶4D traders are more profitable and survive longer in the markets, as was previously reported, but we now find the effect is largely mediated through a higher tolerance for risk.

**Figure 4:**
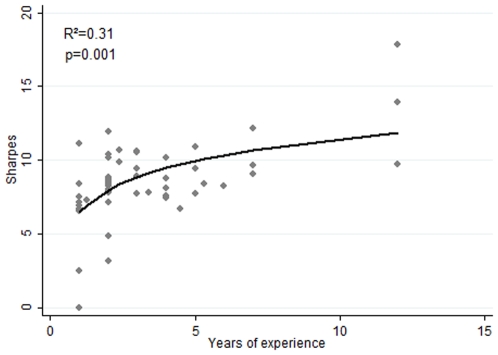
Sharpe Ratio and years of experience. Trader Sharpe Ratios plotted against the number of years they have traded. Sharpe Ratios have been box-Cox transformed. The fitted curve is logarithmic rather than linear. N = 53.

A high appetite for risk may seem insufficient to ensure survival in the markets. Risk taking in high frequency trading without a modicum of skill could just as easily result in reckless and ruinous behaviour as it could higher returns. Perhaps the observed longevity of high risk (low 2D∶4D) traders comes merely from our observing the results of a process that selects traders who take large positions and who are on a lucky streak. Our sample may therefore suffer from survivor bias. However, if this were the case, then our data would show increasing risk with the number of years of trading, but not increasing Sharpe Ratios. Yet our data do show increasing Sharpe Ratios over time. When we regress the 53 traders' Sharpe Ratios against their years of experience, once again using robust regression on Box-Cox transformed data to dampen outlying values, we find a highly significant relationship (R^2^ = 0.31, p = 0.001. Figure 4).

This result suggests either that this cohort of traders learns to make more money per unit of risk as they gain experience [13], or that the market selects for high Sharpe traders. If the former were the case then over time individual traders, as they learn, would display increasing Sharpes; while if the latter were the case then over time traders would display static Sharpes but low Sharpe traders would drop out of our sample. To test these two possibilities we looked at a subset of 27 traders who shared the same 20 months of P&L. We divided this 20 month period into four sub-periods of five months each, calculated Sharpes for each sub-period, and then looked at the evolution of each trader's Sharpe [14]. We found that between the first five month period and the last the average Sharpe ratio increased by 0.70 (Table 1), a result shown to be highly significant by a repeated measures ANOVA (F(3, 78) = 4.15, p = 0.0087). Our data thus suggest that Sharpe Ratios increase over time because traders learn to make more money per unit of risk they take.

**Table 1:** Evolution of individual Sharpe Ratios over a 20-month period.

Months	Average Sharpe	Average increase relative to 1^st^ 5 months
1–5	0.840	0.000
6–10	1.367	0.526
11–15	1.352	0.511
16–20	1.539	0.699

Average Sharpe Ratios calculated for four subsequent periods of five months each. Sharpe ratios increased significantly over the 20 months suggesting that traders were learning how to make more money per unit of risk taken.

## Discussion

The Efficient Market Hypothesis claims that traders and asset managers cannot beat the market. Market prices move randomly so the expected return from trying to buy low and sell high should be zero, less transactions costs. Invested capital, on the other hand, will on average show positive returns and these returns should display i) a linear relationship with the investment's risk; and ii) a Sharpe Ratio equal to that of the broad market. We have found a cohort of experienced high frequency traders who consistently make money trading the market and who attain Sharpe Ratios higher than the Dax, their benchmark index.

The fact that these traders make any money at all trading a supposedly random market is enough, according to some economists, to present anomalous data for EMH. Robert Shiller, for one, interprets EMH this way. He further claims that EMH implies that there can be no return to intelligence, education, training, persistence or any other trait normally associated with success in an activity [15]. Experienced investors, therefore, should not outperform beginners, and neither should outperform the dart board. However, Shiller cites a study of profitable day traders on the Taiwan Stock Exchange as evidence counting against this view [16]. Other studies also find evidence of the persistence of trading profits among a cohort of traders [17], [18]. Harris and Schultz find evidence that small, independent high frequency equity traders consistently make money trading with larger and better informed dealers; and they surmise that these traders can do so because they keep a larger percentage of their profits, giving them greater motivation to pay attention to small price discrepancies [17] (see also [19]). Barber *et al* find evidence that successful traders make money because they are faster at responding to information [16], a possibility we too considered in our previous 2D∶4D study when looking at possible effects of androgens on speed of reactions [7].

These studies, as well as our own, showing the existence of consistently profitable traders, do seem inconsistent with the claims of EMH. However, a proponent of EMH could always counter that the successful traders are in the same position as a coin flipper who has just flipped 20 heads in a row – one would expect this lucky streak to end. One could say the same about traders with Sharpe Ratios higher than the broad market. Yet the existence of a highly significant relationship between the traders' years of experience and their Sharpe Ratios suggests strongly that the performance of this cohort of traders is not due to chance. Furthermore, the increase over time of individual Sharpe Ratios suggests that traders are learning to take better risks. This learning, it should be added, could be due to both individual effort and effective training and management on the part of the employing firm.

The results presented here may conflict with the assumptions of the Efficient Markets Hypothesis, but they accord with common sense. Traders are risk takers so need a high tolerance for risk, a trait predicted by a measure of prenatal androgen exposure. However, this trait, like height or speed in sports, may count for little without proper training and management. In trading, as in sports, biology needs the guiding hand of experience.

### Postscript

It is common for traders and fund managers to boast high returns and Sharpe Ratios during bull markets only to have their excess returns disappear in the next bear market. The credit crisis that began in 2007, just after the end of our study, put an end to countless such claims of out-performance, with many banks and hedge funds losing more money in 2008 than they had made in the previous five years. We wondered if our traders had suffered the same fate. We therefore asked the trading managers at our study firm how their traders had performed in 2008 relative to their average P&L between 2005–2007. The managers provided data showing that the experienced traders remaining at the firm during 2008 (n = 22) made on average more money than they had during the study, with many of them having record years.

In trying to account for the differing fate of traders at our study firm, on one hand, and at many of the banks and hedge funds, on the other, it is worth pointing out that these traders differ in one important respect – their compensation schemes. Bankers and hedge fund traders are awarded a yearly bonus, one amounting to as much as 20% of P&L. Importantly, their bonus each year is independent of previous years, meaning that a trader could in principle make $100 million a year for four years, receive a yearly bonus of $20 million, and on the fifth year lose $500 million and receive no bonus. After 5 years he has lost the bank $100 million but has pocketed a total of $80 million in bonuses and does not have to give them back. Such a compensation scheme gives traders a strong incentive to maximize the variance of their P&L and the frequency of payouts. This strategy increases their chances of being paid at what are called ‘high-water marks’, like the years when the trader made $100 million. Such a compensation scheme, in short, rewards risk rather than Sharpe Ratios.

It is possible, therefore, that banks and to a lesser extent hedge funds attract traders with an appetite for large amounts of risk rather than long term prudence. Our traders, on the other hand, have no year end bonus; they have only profit sharing, so if they lose money for the firm they lose it for themselves. These traders have, therefore, a strong incentive to lower, not raise, their variance.

## Materials and Methods

### Subjects

We recruited 53 male traders from a trading floor in the City of London which employed approximately 250 traders. Recruitment was conducted by means of an introductory note explaining that we were looking at the effects of prenatal testosterone on the shape of the participant's right hand. Traders were informed that they would receive a summary of our findings, but were not offered payment. All subjects completed a short questionnaire asking their age, years of trading, P&L history, number of older brothers, and whether they had broken the index or ring finger of their right hand. They also signed an informed consent form. All handprints, questionnaire data, and P&L from the bank were coded by an independent laboratory technician in Cambridge. The study was approved by the Ethics Committee of the School of Biological Sciences at the University of Cambridge.

### P&L Data

The employing firm provided for each trader 20 months of monthly P&L data, net of broker commissions. P&L is recorded by the back office accounting system so is free of reporting bias. During the study, running from the beginning of 2005 through autumn 2007, some traders left the firm while others joined, so for some traders we had less than 20 months of P&L data and the dates of their samples varied. There were, for instance, fewer observations in 2007 than in 2005–2006. To make sure that a difference between the average Sharpe Ratio of this sample of traders and the market is not driven by the specific dates at which our traders are observed, we computed the market Sharpe by weighting the market monthly returns with the number of traders observed each month (Note S1).

It has been reported that Sharpe Ratios can be manipulated by traders through the use of options contracts [8]. However, the traders at this firm could not do so because they did not position options.

### 2D∶4D Measurements

A subset of our traders, n = 44, took part in a previous study looking at 2D∶4D and P&L. Our procedure for measuring digit ratio is described in Coates *et al*, 2009 [7].

### Statistics

The right-skew in the P&L and Sharpe Ratio data was corrected by a Box-Cox transformation (SI). 2D∶4D data approximated a normal distribution. To dampen the effect of extreme data points we employed either robust regression on Box-Cox transformed variables or the more conservative bootstrap techniques applied to raw data. Our statistical methods are more fully described in SI. Statistical analyses were performed with Stata, release 10/SE (Stata).

## Supporting Information

Note S1 A note on trader Sharpe Ratio.
(0.75 MB DOC)
