# Supplementary material for: A Note on Trader Sharpe Ratios
Source: PLoS One. 2009 Nov 25;4(11):e8036. doi: 10.1371/journal.pone.0008036 (PMC2776986; doi:10.1371/journal.pone.0008036)
Supplement: Note S1 — A note on trader Sharpe Ratio. (0.75 MB DOC) [file pone.0008036.s001.doc]

**Supplementary Information: A note on trader Sharpe Ratios**

**John M Coates, Lionel Page**

**Measure of the Sharpe Ratio for the broad market (Dax)**

To measure the Sharpe Ratio of the market, we used the monthly rate of return of the Dax during the period of observation of the traders’ activity. We used the 1yr German government bond rate as the risk free rate over the period. The monthly Sharpe Ratio of the Dax is then calculated as:

Where *R* is the monthly return and *R_f_* the risk free rate of return during the corresponding month. Our traders were observed from Oct 2004 to Oct 2007 with the period of observations varying between traders. Our observations are more concentrated on 2005-2006 with less traders observed in 2007. To make the Dax Sharpe comparable to the traders’ Sharpe for the period, we computed the average rate of the Dax’s net return *E*[*R-R_f_*] as a weighting average using the number of observations per month in our trader sample as weights. Trader Sharpe Ratios were calculated as P&L divided by standard deviation of P&L because the rate they earned on their capital less the risk free rate netted close to zero, as explained in the main paper.

In order to check the robustness of our result we also measured the Sharpe of the Dax in pounds (as the traders’ P&L is reported in pounds sterling and they maintain their capital in sterling) taking into account the changes in the euro/pound exchange rate over the study period. Such a calculation is motivated by the fact that we measure the traders’ profit and losses in pounds. To do this calculation we converted the value of the Dax index every day using the daily euro/pound exchange rate and we did compute the Sharpe on this new series using the same method. We used the 1yr Gilt rate (rate of UK government bonds) as the risk free rate in the UK. We found a slightly smaller Sharpe (0.377) mainly due to the fact that the pound lost some of its value over the period relative to the euro, and also partially due to the fact that UK rates are slightly higher than in the Eurozone. As this Sharpe ratio is lower than the non-currency adjusted Sharpe, we kept the original Sharpe in our calculation to adopt a conservative approach.

We also measured the Sharpe of the Bund market (German government bonds) over the period. The monthly Sharpe of the Bund market is negative over the period of observation (-0.03). When weighting each month to take into account the distribution of our observation on the period, the Sharpe of the Bund market is slightly positive (0.11), but still much smaller than the Dax monthly Sharpe . Here again the Sharpe ratio is lower than the one we computed for the Dax. To be conservative we used the Dax Sharpe ratio as a measure of the broad market Sharpe.

**The Box–Cox Transformation of the Average P&L. SD P&L and Sharpe Ratio.**

A skewness test of normality indicates that the average P&L, SD P&L and Sharpe Ratio are not normally distributed (while the 2D:4d ratio is).

| **Skewness tests for Normality** | |
| --- | --- |
| Variable | Pr(Skewness) |
| SD P&L | <0.001 |
| Mean P&L | <0.001 |
| Sharpe | <0.001 |
| 2D:4d | 0.34 |

We normalised these three variables using a Box-Cox transformation. For each of these variables, the Box–Cox transformation [[[1]](#endnote-2)] is obtained by first adding a constant to the average P&L to make all values positive, and then estimating by maximum likelihood the value of the power so that the skewness of the Box–Cox power transformation is zero.

**Bootstrap test of Sharpe Ratios**

In order to take into account the fact that the Sharpe Ratio of the market can be considered as the realisation of a random variable with an underlying distribution, we used a resampling technique (boostrap) to create a distribution of the Sharpe Ratio of the market. New samples of monthly returns of the Dax were created by bootstrap [[[2]](#endnote-3)] with sampling probabilities for each month proportional to the number of observations in our sample, to control for the fact that the observations in our sample are not uniformly distributed over the period. For each of the samples formed, a Sharpe Ratio is computed. As we are interested in the difference between the traders we want to compare these new market Sharpes to the traders’ average Sharpe. To do so we also need to take into account the fact that the observed average Sharpe of the traders is also the realisation of a random variable. We use again bootstrap to create different samples of traders’ P&L by resampling their months of observation, and we compute an average Sharpe for each of these samples. We ran these bootstraps in parallel and each time measured the difference between the market Sharpe and the traders’ average Sharpe. We used 10000 bootstrap replications to estimate the distribution of the difference between the traders and the market Sharpes. From this distribution we can estimate an average difference and a standard error which allows us to compute a test of difference to zero (parametric bootstrap).

| **Test for the Sharpe of the Dax** | | | | |
| --- | --- | --- | --- | --- |
| Market Sharpe ratio: 0.534 | | | | |
|  | Average  difference in Sharpes  (traders-markets) | Bootstrap Std. Err. | z | *p* value |
| All traders | 0.166 | 0.254 | 0.65 | 0.516 |
| Inexperienced  Traders | -0.144 | 0.404 | -0.36 | 0.719 |
| Experienced traders | 0.486 | 0.226 | 2.15 | 0.032 |

| **Test for the Sharpe of the Dax (returns denominated in pounds)** | | | | |
| --- | --- | --- | --- | --- |
| Market Sharpe ratio: 0.377 | | | | |
|  | Average  difference in Sharpes  (traders-markets) | Bootstrap Std. Err. | z | *p* value |
| All traders | 0.323 | 0.298 | 0.63 | 0.278 |
| Inexperienced  Traders | -0.013 | 0.333 | -0.04 | 0.968 |
| Experienced traders | 0.643 | 0.198 | 3.25 | 0.001 |

**Regression of Sharpe on years: OLS with robust matrix of variance covariance**

| Number of obs | F( 1, 51) | Prob > F | R-squared |  |  |  |
| --- | --- | --- | --- | --- | --- | --- |
| 53 | 12.69 | 0.0008 | 0.3118 |  |  |  |
|  |  |  |  |  |  |  |
| Sharpes | Coef. | Std. Err. | t | P>t | [95% Conf. | Interval] |
| Log years | 2.171039 | 0.609344 | 3.56 | 0.001 | 0.947729 | 3.394349 |
| Constant | 6.450864 | 0.679921 | 9.49 | 0 | 5.085866 | 7.815861 |

**Regression of P&L on risk, 2D:4D, and experience**: OLS with robust matrix of variance covariance

| Number of obs | F( 3, 43) | Prob > F | R-squared |  |  |  |
| --- | --- | --- | --- | --- | --- | --- |
| 47 | 261.57 | 0 | 0.9421 |  |  |  |
|  |  |  |  |  |  |  |
| Mean P&L | Coef. | Std. Err. | t | P>t | [95% Conf. | Interval] |
| SD P&L | 1.023588 | 0.05175 | 19.78 | 0 | 0.919225 | 1.127952 |
| 2D:4D | 0.177403 | 1.57103 | 0.11 | 0.911 | -2.99088 | 3.345686 |
| Years of experience | 0.048248 | 0.016 | 3.02 | 0.004 | 0.01598 | 0.080515 |
| Constant | -0.34012 | 1.553025 | -0.22 | 0.828 | -3.47209 | 2.791857 |

**Evolution of the Sharpe of each trader over time**

In order to determine if the Sharpe Ratios of traders tend to increase over time, we looked at the traders which we observed on the longest span of time (20 months, 27 traders). We divided the period of observation of these traders into 4 periods of 5 months each. For each of these periods we computed a monthly Sharpe as the ratio of the mean monthly P&L to the standard deviation of their monthly P&L. We then used a repeated measure ANOVA to check if the observed differences are significant. As indicated in the main text of the article there is a significant increase over time of the traders’ Sharpes (P=0.0087). We also used a panel regression with fixed effect and robust standard error to measure if there is an increase of the Sharpe ratios over time for each trader (this procedure is similar to a repeated measure anova but does not impose the same variance for the error terms over each time period). The table below shows the result of this estimation. The coefficient of our time variable (which takes four values 1,2,3,4) is highly significant (P=0.002). It indicates that overall the traders’ Sharpes increased on average by 0.2 every 5 months. This result is not only statistically significant but practically important in terms of magnitude.

**Regression of Sharpe on time: Panel regression with fixed effects and** robust matrix of variance covariance

| Number of units: 27 | F( 1, 80) | Prob > F | R-squared |  |  |  |
| --- | --- | --- | --- | --- | --- | --- |
| Number of observation: 108 | 9.82 | 0.002 | 0.05 |  |  |  |
|  |  |  |  |  |  |  |
| Sharpe ratio | Coef. | Std. Err. | t | P>t | [95% Conf. | Interval] |
| Time period | 0.2084062 | 0.0665074 | 3.13 | 0.002 | 0.0760522 | 0.3407602 |
| Constant | -0.34012 | 0.124424 | 7.73 | 0.000 | 0.71448 | 1.209703 |

The analyses were conducted with Stata (Stata Corp.), Release 10/SE.

**References**

1. Box G, Cox D (1964) An analysis of transformations. J R Stat Soc [Ser B] 26: 211–246. [↑](#endnote-ref-2)
2. Efron, B. Tibshirani, R (1993). An Introduction to the Bootstrap. Chapman and Hall, New York. [↑](#endnote-ref-3)
